# The influence of a swab type on the results of point-of-care tests

**DOI:** 10.1186/s13568-020-00978-9

**Published:** 2020-03-12

**Authors:** Aleksandra Anna Zasada, Katarzyna Zacharczuk, Katarzyna Woźnica, Małgorzata Główka, Robert Ziółkowski, Elżbieta Malinowska

**Affiliations:** 1 Department of Sera and Vaccines Evaluation, National Institute of Public Health-National Institute of Hygiene, Warsaw, Poland; 2 Department of Bacteriology and Biocontamination Control, National Institute of Public Health-National Institute of Hygiene, Warsaw, Poland; 3 Faculty of Chemistry, The Chair of Medical Biotechnology, Warsaw University of Technology, Warsaw, Poland; 4 Centre for Advanced Materials and Technologies CEZAMAT, Polna 50, 00-644 Warsaw, Poland

**Keywords:** POCT, Swabs, Clinical diagnostics, Sample collection

## Abstract

Most point-of-care tests (POCT) use swabs for sampling and/or for applying a sample on the test. A variety of swabs differing in tip materials is commercially available. Different tip materials have different chemical and physical characteristics which might influence the specimen collection and release. We investigated properties of various types of swabs used in clinical diagnostics with focusing on two kinds of analytes, DNA and proteins, which are most often used targets in POCT. As the model samples we used diphtheria toxoid NIBSC 69/017 for investigating recovery of protein analytes such as antigens and bacterial strains of *Escherichia coli* ATCC 25922, diphtheria toxin-producing *Corynebacterium diphtheriae* NCTC 10648, and the clinical isolate nontoxigenic *C. diphtheriae* 5820/15 for investigating the recovery of nucleic acids. We investigated four types of swabs most commonly used in clinical diagnostics in terms of absorption capacity and efficiency of release of nucleic acids and proteins. Volume uptake was measured in milligrams. For DNA release various washing out buffers were used and the amount of released DNA was measured spectrophotometrically. The amount of protein released from the swabs were examined using the Lowry assay. We observed statistically significant differences (*p *< 0.05) in the mean weights of absorbed liquid, in the DNA recovery and protein recovery by the four variety of swab examined. However, the efficiency of DNA and protein release was not correlated to the absorbed volume of a sample, but rather to the properties of swabs. The swab composition and structure can have a significant impact on the collection and release efficiency of a sample. Therefore, validation of POCT in relation to the used swabs for sampling is really important. The use of inappropriate swabs could lead to false negative or misleading analysis results.

## Introduction

In diagnostics, the development of point-of-care tests (POCT) is receiving considerable attention. Usually, the validation of POCT is focused on various types of samples and matrices such as blood, serum, sputum, urine, nose swabs, throat swabs and wound swabs, as well as the anatomical sites to be sampled (e.g. Maffert et al. 2017; Rozand 2014; Senn et al. 2012). Most POCT use swabs for sampling and/or for applying a sample on the test; however, usually the validation of POCT with various swabs is not usually performed. Swabs commercially available can differ in tip materials, such as nylon, rayon, cotton, polyester, polyurethane, calcium alginate and the chemical or physical characteristics can influence the specimen collection and release; moreover also the structure of the tips can vary (i.e. flocked fiber, tightly wound and knitted). Most studies related to various types of swabs have focused on environmental samples and collection of samples from different types of surfaces (e.g. Dadhania et al. 2013; Hansson et al. 2009; You et al. 2019). In clinical diagnostics, the evaluation of swabs was performed mainly based on CFU which provides information only about living bacterial cells (Dube et al. 2013; Warnke et al. 2014a, b). Usually POCT are not used for cultivated microorganisms, but more often for the detection of analytes such as nucleic acids and antigens.

In this study, we investigated the properties of different swabs used in clinical diagnostics and specifically focused on two types of analytes, i.e., DNA and proteins, which are regular targets for POCT.

## Materials and methods

### Model samples

In this study we used two types of model samples: (i) diphtheria toxoid NIBSC 69/017 for investigating recovery of protein analytes such as antigens and (ii) three bacterial strains at different concentrations, i.e., *Escherichia coli* ATCC 25922, diphtheria toxin-producing *Corynebacterium diphtheriae* NCTC 10648, and the clinical isolate nontoxigenic *C. diphtheriae* 5820/15 isolated from blood, for investigating the recovery of nucleic acids.

### Swabs

**Fig. 1:**
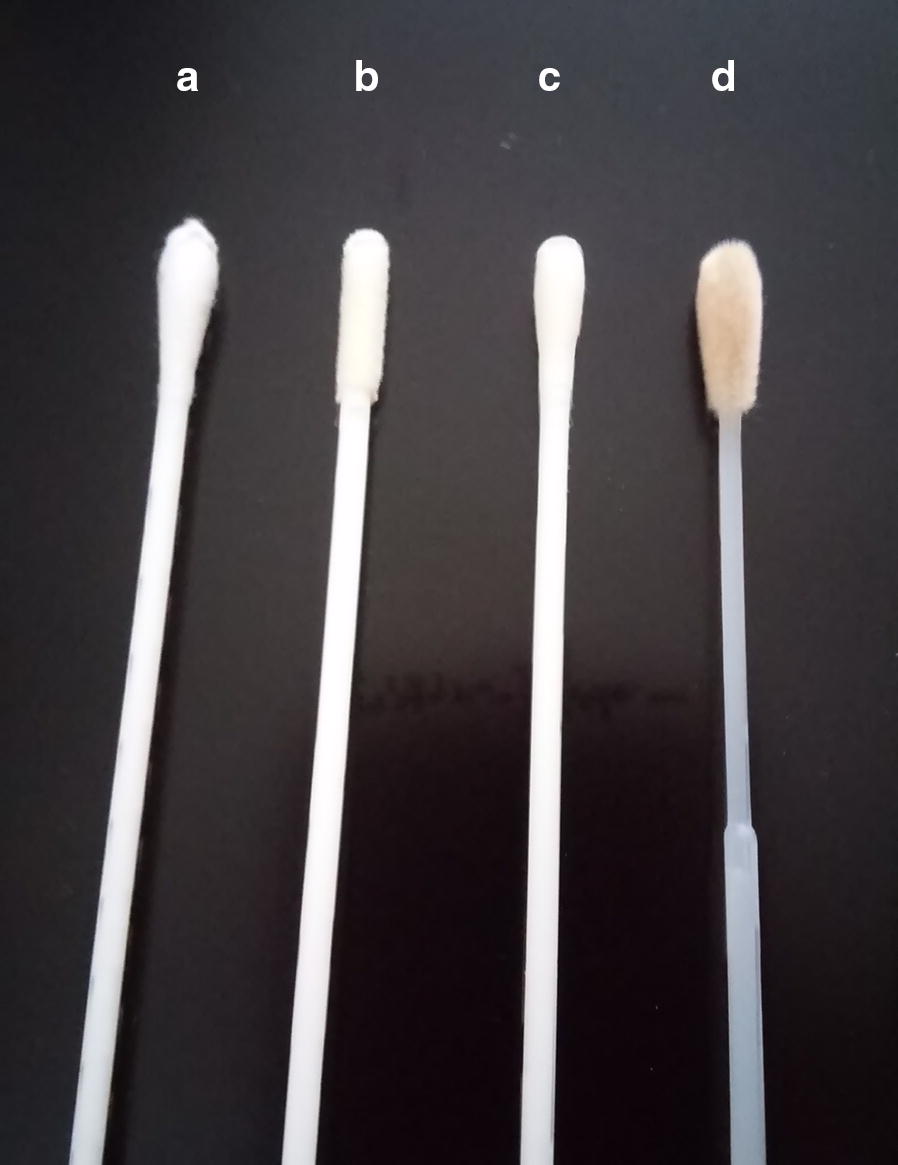
Swab types used in the study: A—dacron swab, B—polyurethane foam, C—rayon swab, D—flocked nylon swab

We investigated four different commercially available swabs: FLOQSwabs (Copan Italia S.p.A, Italy), which are flocked swabs made of nylon; rayon swabs (Copan Italia S.p.A, Italy); dacron swabs (Copan Italia S.p.A, Italy); and BBL Culture Swabs (Becton, Dickinson and Company, USA), which are swabs composed of polyurethane foam (Fig. 1).

### Measurement of the absorbed volume

We transferred 500 µl of water to an Eppendorf tube, and then the tube was weighted using a balance Discovery (Ohaus, Germany). The swab was immersed in water for 5–10 s. After removing the swab, the tube was weighted. The weight of absorbed liquid was calculated as a difference between the weight of the tube before swab immersion and after removing the immersed swab. Furthermore, the weight of water absorbed by a swab (m) was converted into volume (v) using the following calculation: v = m/d, where d is density of water (1 kg/m^3^). The experiment was repeated five times for each type of swab.

### Measurement of DNA recovery

In the preliminary study, DNA was extracted from suspensions of the three above mentioned bacterial strains using Wizard Genomic DNA Purification Kit (Promega, Germany) according to the manufacturer’s protocol. A 24-h culture of bacterial strains on Columbia agar with 5% sheep blood (BioMerieux, France) was suspended in saline solution at appropriate densities. We used ten different densities (from 0.5 McF to 9 McF) of the bacterial suspension, and the amount of extracted DNA was measured using a BioPhotometer^®^ model 6131 (Eppendorf, Germany). The extraction was triplicated for each suspension and for each bacterial strain. Based on the results of the pilot study, we selected both the bacterial strains and density of the suspension most suitable for further experiments.

We used nine types of buffers to wash out bacterial cells and DNA from tested swabs: phosphate-buffered saline (PBS), tris-EDTA buffer (TE), molecular grade water, AL buffer (Qiagen), ATL buffer (Qiagen), lysis buffer (Qiagen), saline, 0.5% Tween 20 and Nucleic Lysis Solution (Promega). We immersed the swabs in a bacterial suspension and manually agitated for ~ 10 s. Then, the swabs were transferred into 200 µl of each different buffer and manually agitated for ~ 10 s to release bacterial cells and DNA. Next, the swab was removed and the obtained suspension was used for DNA extraction using the Wizard Genomic DNA Purification Kit (Promega), according to the manufacturer’s instruction. All the experiments were performed six times for each combination swab/bacterial strain/type of buffer, by two different laboratory workers. We used the bacterial suspension in a volume equal to average volume absorbed by particular type of swabs as a positive control, and the amount of extracted DNA was measured using a BioPhotometer^®^ model 6131 (Eppendorf, Germany).

### Measurement of protein recovery

We used the concentration of 2 µg/ml of diphtheria toxoid for the test. We transferred 500 µl of diphtheria toxoid suspension to an Eppendorf tube. Every swab was immersed into the suspension for 5–10 s, and then transferred to an Eppendorf tube containing 500 µl of PBS and manually agitated for 5–10 s and removed. The amount of protein released from the swabs was examined using the Lowry assay according to European Pharmacopoeia (Ph. Eur. 2.5.33; 01/2008:20533). We used eight reference solutions of diphtheria toxoid to prepare the standard curve. For calculating the standard curve, we plotted the absorbance of the reference solutions against the protein concentrations along with linear regression. We determined the concentrations of the diphtheria toxoid in test solutions using a standard curve. The experiment was performed three times for each type of swabs and each concentration of diphtheria toxoid.

### Statistical analysis

The arithmetic mean and standard deviations were calculated using Excel. Statistical analysis was performed using the Kruskal–Wallis test, which is suitable for comparing two or more independent samples of the same or different size. The results were regarded as significant at a *p* value of < 0.05.

## Results

**Fig. 2:**
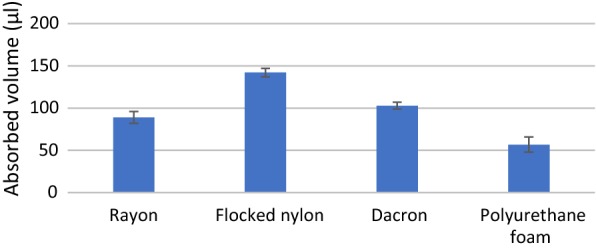
The volume of water absorbed by investigated types of swabs

Statistically significant differences (*p *< 0.05) were observed in the mean weights of absorbed liquid by the four types of analysed swabs (Table 1). The calculated *p* value was 0.0006, and the calculated mean volumes of absorbed liquid were 90 µl, 142 µl, 103 µl and 57 µl for rayon swab, flocked nylon swab, dacron swab and polyurethane foam swab, respectively (Fig. 2).

**Table 1:** Weight of water absorbed by various types of swabs (g)

Experiment	Swab type			
	Rayon	Flocked nylon	Dacron	Polyurethane foam
1	0.08889	0.14385	0.10770	0.04498
2	0.09027	0.13655	0.10454	0.05731
3	0.10127	0.14943	0.10123	0.05126
4	0.08063	0.14460	0.09720	0.06365
5	0.08317	0.13577	0.10645	0.07043
Mean ± SD	0.088846 ± 0.007	0.14204 ± 0.005	0.103424 ± 0.004	0.057481 ± 0.009

*SD* standard deviation

We investigated DNA recovery and protein recovery from the four types of swabs rather than CFU because POCT are usually not focused on cultivating microorganisms but detecting genetic markers and protein markers such as antigens and antibodies.

In the preliminary study for the DNA recovery examination, we compared DNA extraction efficiency using various densities of suspensions of three bacterial strains: *E. coli* reference strain, toxigenic *C. diphtheriae* reference strain and non-toxigenic *C. diphtheriae* clinical isolate. In this experiment we did not use any swabs. The results are presented in Additional file 1: Table S1. For further studies with the various types of swabs the two *C. diphtheriae* strains were selected as the most challenging for DNA extraction, and the suspension of 9 McF.

**Fig. 3:**
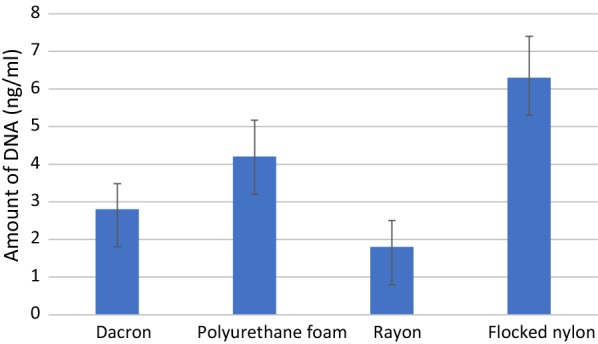
Amount of DNA recovered from investigated types of swabs

**Fig. 4:**
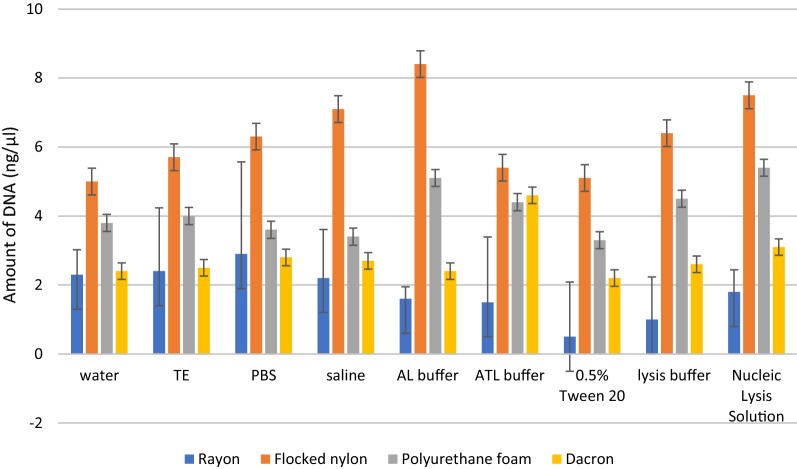
DNA recovery using various washing out buffers and various types of swabs

Note that DNA recovery was strongly related to the type of swab and statistically significant differences (p < 0.05) were observed among types of swabs regardless of the buffer used (*p* value = 0.00001) (Fig. 3). We observed significant differences among buffers used for washing out the bacterial cells only for rayon swabs (*p* value = 0.000390). We obtained the highest amount of DNA for flocked nylon swabs, and the worst DNA recovery was obtained for rayon swabs combined with 0.5% Tween20 as a washing out buffer (Fig. 4). Table 2 shows the average amount of DNA (ng/ml) extracted using four types of swabs combined with various washing out buffers.

**Table 2:** Average amount of DNA (ng/ml) extracted using four types of swabs combined with various washing out buffers

Type of swab	Washing out buffer									Mean ± SD
	Water	TE	PBS	Saline	AL buffer	ATL buffer	0.5% Tween 20	Lysis buffer	Nucleic lysis solution	
Rayon	2.3	2.4	2.9	2.2	1.6	1.5	0.5	1	1.8	1.8 ± 0.702
Flocked nylon	5	5.7	6.3	7.1	8.4	5.4	5.1	6.4	7.5	6.3 ± 1.095
Polyurethane foam	3.8	4	3.6	3.4	5.1	4.4	3.3	4.5	5.4	4.2 ± 0.968
Dacron	2.4	2.5	2.8	2.7	2.4	4.6	2.2	2.6	3.1	2.8 ± 0.679

*SD* standard deviation

**Fig. 5:**
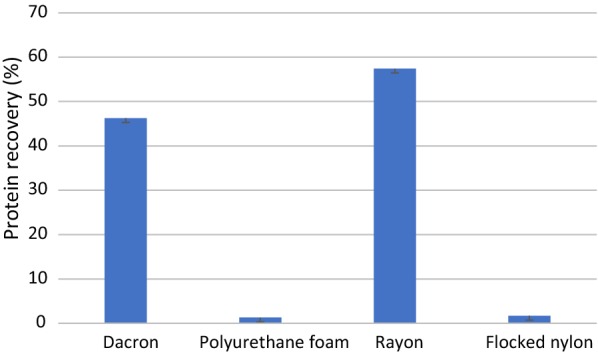
Diphtheria toxoid recovery from investigated types of swabs

Interestingly, the recovery of protein from the swabs was unrelated to the absorbed volume of liquid. The most efficient protein recovery was measured for rayon swabs and dacron swabs (Table 3). Protein recovery from flocked nylon swabs and polyurethane foam swabs was > 30 times lower (Fig. 5).

**Table 3:** Diphtheria toxoid recovery from investigated types of swabs (µg/ml)

Experiment	Swab type			
	Rayon	Flocked nylon	Dacron	Polyurethane foam
1	59.022	1.859	46.416	1.316
2	57.066	1.642	46.198	1.207
3	56.196	1.642	46.198	1.424
Mean ± SD	57.428 ± 1.447	1.714 ± 0.125	46.271 ± 0.125	1.316 ± 0.154

*SD* standard deviation

## Discussion

In clinical diagnostics, preanalytical process could largely change the sensitivity of a diagnostic assay. Therefore, sample collection quality is crucial for the quality of subsequent analytical tests, and the swab used for sampling can be one of critical points of POCT. Currently, a range of swab types is commercially available. The first cotton swab was developed by the pathologist William Thomas Councilman in 1893 (Councilman 1893). When it was known that wrapped cotton fiber contains inhibitory fatty acids, other materials for producing swab tips were used. Calcium alginate fiber was inhibitory to PCR and toxic for tissue culture; therefore, non-toxic synthetic fiber wrapped swabs, such as dacron and rayon, were used. In 1992, Dickinson patented the non-toxic polyurethane foam-tipped swabs, and Copan patented flocked swabs in 2004 (Rapid Microbiology 2010). In our study, we compared dacron, rayon, polyurethane foam and flocked nylon swabs and excluded cotton and calcium alginate swabs because they are not recommended for microbiological diagnostics (Centers for Disease Control and Prevention 2017, Cloud et al. 2002).

Absorption is considered as a key parameter of sampling swabs (Harry and Madhusudhan 2014; Panpradist et al. 2014). In our study, the evaluated swabs revealed significant differences in the ability to absorb water; however, this parameter was poorly related to the ability of releasing an analyte present in the sample. The highest absorption was revealed for flocked nylon swabs, which are the most efficient for DNA extraction and bacterial culture (Dadhania et al. 2013; Dube et al. 2013; Warnke et al. 2014b). The DNA extraction efficiency for flocked nylon swabs was 3.5 times higher than from rayon swabs in our study, consistent with other past studies Hernes et al. (2011) in which was tested the efficiency of viral DNA extraction from clinical samples comparing flocked nylon swabs and rayon swabs. Interestingly, among the examined swabs, the DNA extraction efficiency was comparable between flocked nylon swabs which showed the highest absorption capacity and polyurethane foam swabs which showed the lowest absorption capacity. However, diphtheria toxoid recovery from flocked nylon swabs was 33.5 times lower that from rayon swabs. Moreover, it was even much more lower than the diphtheria toxoid recovery obtained from polyurethane foam swabs, which was 43.6 times lower than from rayon swabs. Note that the diphtheria toxoid recovery efficiency was comparable for both rayon swabs and dacron swabs.

The recovery of an analyte from a swab might be related to the structure of the swab tip and unspecific interactions between an analyte and the swab material. The structure of polyurethane foam swabs is based on a hydrophobic open cell foam, which limits the volume of sample collected. However, it stays on the surface for easy elution.

The recovery of living bacterial cells, investigated by other researchers, was usually more efficient from flocked nylon swabs and polyurethane foam swabs compared to rayon and dacron swabs (Panpradist et al. 2014; Warnke et al. 2014b). Rayon and dacron swabs are fiber-wrapped swabs, which are hydrophilic but with poor release characteristic because a sample is trapped within the fiber matrix (Dube et al. 2013; Hedin et al. 2010). Unlike bacterial cells experiments, the release of proteins such as diphtheria toxoid was much more efficient from rayon and dacron swabs compared to flocked nylon and polyurethane foam swabs. The flocked swab has been designed for the uptake of a large volume of liquid sample, which stays close to the surface and elutes out rapidly and spontaneously (Rapid Microbiology 2010). This assumption can be true for water; however, the absorption and release of liquid might not be related to the release of a specific analyte present in the liquid.

It is supposed that DNA extraction methods might influence the DNA recovery from different swabs (Brownlow et al. 2012). However, we could not confirm the influence of the various buffers used to wash samples from swabs for DNA recovery. Significant differences among DNA extraction efficiency using various washing out buffers were observed only when samples were collected with rayon swabs. Unlike our results, You et al. (You et al. 2019) observed significantly greater amount of extracted DNA when a sample was washed out with buffer containing 1% Tween 20 and 1% glycerol in PBS in comparison to PBS and GS commercial solution. In our study, PBS was the best buffer for washing out the samples from rayon swabs for DNA extraction, whereas washing out the samples using 0.5% Tween 20 resulted in lower DNA extraction efficiency among all tested buffers.

Results of our study emphasizes the importance of validation of POCT in terms of swab types used for sample collection because commercially available types of swabs differ significantly in their properties. There is no universal type of swabs and therefore the swab type should be selected and evaluated with regard to an analyte for specific POCT. The swab composition and structure can have a significant impact on collection and release efficiency and the use of inappropriate type of swab could lead to false negative results or to a lower detection limit in samples.

## Supplementary information

**Additional file 1: Table S1.** Average amount of extracted DNA (ng/µl).

## Data Availability

The data of this research are inserted in the present article; other data is available if needed.

## Abbreviations

POCT: Point-of-care tests
PBS: Phosphate-buffered saline (PBS)
TE: Tris–EDTA buffer
