# Supplementary material for: The influence of a swab type on the results of point-of-care tests
Source: AMB Express. 2020 Mar 12;10:46. doi: 10.1186/s13568-020-00978-9 (PMC7067933; doi:10.1186/s13568-020-00978-9)
Supplement: Supplementary file 1 — Additional file 1: Table S1. Average amount of extracted DNA (ng/µl). [file 13568_2020_978_MOESM1_ESM.docx]

Additional file

**Influence of a swab type on the results of point-of-care tests**

Aleksandra Anna Zasada^1^, Katarzyna Zacharczuk^2^, Katarzyna Woźnica^1^, Małgorzata Główka^1^, Robert Ziółkowski^3^, Elżbieta Malinowska^3,4^.

^1^National Institute of Public Health - National Institute of Hygiene, Department of Sera and Vaccines Evaluation, Warsaw, Poland

^2^National Institute of Public Health - National Institute of Hygiene, Department of Bacteriology and Biocontamination Control, Warsaw, Poland

^3^Warsaw University of Technology, The Chair of Medical Biotechnology, Faculty of Chemistry, Warsaw, Poland

^4^Centre for Advanced Materials and Technologies CEZAMAT, Polna 50, 00-644 Warsaw, Poland

Corresponding author:

Aleksandra Anna Zasada

e-mail: [azasada@pzh.gov.pl](mailto:azasada@pzh.gov.pl)

phone: +48 22 5421212

fax: +48 22 8497484

Additional Table S1. Average amount of extracted DNA (ng/µl).

| Density (McF) | *E. coli* ATCC 25922 | *C. diphtheriae* 5820/15 (tox -) | *C. diphtheriae* NCTC 10648 (tox +) |
| --- | --- | --- | --- |
| 0.5 | 22.0 | 3.6 | 1.2 |
| 1 | 22.4 | 5.4 | 2.7 |
| 2 | 22.4 | 5.3 | 5.5 |
| 3 | 30.7 | 6.2 | 8.1 |
| 4 | 58.0 | 8.6 | 7.2 |
| 5 | 70.0 | 8.8 | 7.6 |
| 6 | 62.8 | 13.1 | 8.7 |
| 7 | 87.6 | 22.6 | 10.0 |
| 8 | 95.3 | 22.8 | 17.6 |
| 9 | 103.3 | 39.6 | 32.5 |
